# Microbiota Controls the Homeostasis of Glial Cells in the Gut Lamina Propria

**DOI:** 10.1016/j.neuron.2014.12.037

**Published:** 2015-01-21

**Authors:** Panagiotis S. Kabouridis, Reena Lasrado, Sarah McCallum, Song Hui Chng, Hugo J. Snippert, Hans Clevers, Sven Pettersson, Vassilis Pachnis

**Affiliations:** 1 William Harvey Research Institute, Queen Mary University London, London EC1M 6BQ, United Kingdom; 2 Division of Molecular Neurobiology, MRC National Institute for Medical Research, The Ridgeway, Mill Hill, London NW7 1AA, United Kingdom; 3 Lee Kong Chian School of Medicine and School of Biological Sciences, Nanyang Technological University, 50 Nanyang Avenue, Singapore 639798, Singapore; 4 Department of Microbiology, Tumor and Cell Biology, Karolinska Institute, 17177 Stockholm, Sweden; 5 Hubrecht Institute – KNAW and University Medical Centre Utrecht, 3584 CT Utrecht, The Netherlands

## Abstract

The intrinsic neural networks of the gastrointestinal tract are derived from dedicated neural crest progenitors that colonize the gut during embryogenesis and give rise to enteric neurons and glia. Here, we study how an essential subpopulation of enteric glial cells (EGCs) residing within the intestinal mucosa is integrated into the dynamic microenvironment of the alimentary tract. We find that under normal conditions colonization of the lamina propria by glial cells commences during early postnatal stages but reaches steady-state levels after weaning. By employing genetic lineage tracing, we provide evidence that in adult mice the network of mucosal EGCs is continuously renewed by incoming glial cells originating in the plexi of the gut wall. Finally, we demonstrate that both the initial colonization and homeostasis of glial cells in the intestinal mucosa are regulated by the indigenous gut microbiota.

## Highlights

• The glial cell network of the gut mucosa develops after birth
• Mucosal glial cells are a continuously renewed homeostatic cell population
• Microbiota regulates the development and homeostasis of mucosal glial cells

## Teaser

Mucosal glial cells are important for maintaining the intestinal epithelial barrier and regulating immune responses in the gut. Kabouridis et al. demonstrate that signals from microbiota regulate the postnatal development and the continuous renewal of this protective cell network.

## Introduction

Glial cells encompass diverse neuroectodermal cell populations that are essential for the organization and function of the nervous system (Verkhratsky and Butt, 2007). In addition to their roles in providing support and nourishment for neurons, glial cells regulate synaptic transmission (Clarke and Barres, 2013), maintain the blood-brain barrier (Alvarez et al., 2013), and mediate communication between the nervous and immune system (Jensen et al., 2013). Consequently, glial cell deficits are associated with developmental, degenerative, and inflammatory disorders of the nervous system (Skaper et al., 2014). The enteric nervous system (ENS) encompasses the intrinsic neural circuits of the gastrointestinal tract (GI), which are organized into a vast network of interconnected ganglia distributed into two concentric layers within the gut wall, the outer myenteric (MP) and the inner submucosal (SMP) plexus (Furness, 2006). The ENS regulates most aspects of GI physiology, such as peristalsis, blood supply to the gut wall, and secretion (Furness, 2006), and constitutes a relay station in the bi-directional neuro-endocrine pathways that connect the digestive system and the brain (gut-brain axis) (Collins et al., 2012). In rodents, enteric neurons are born during embryogenesis and early postnatal life and are restricted to the ganglia (Laranjeira et al., 2011; Liu et al., 2009; Pham et al., 1991). Enteric glial cells (EGCs) outnumber enteric neurons by 4:1 and are located within ganglia and extraganglionic sites, including the smooth muscle layers and the intestinal mucosa (Boesmans et al., 2015; Gershon and Rothman, 1991; Gulbransen and Sharkey, 2012; Rühl, 2005). In contrast to enteric neurogenesis, low levels of gliogenesis have been observed in enteric ganglia of unchallenged adult rodents, although the destination of the newly generated glial cells and their function remains unclear (Joseph et al., 2011). Based on morphological features and location, EGCs are subdivided into distinct subtypes that share molecular and functional characteristics (Boesmans et al., 2015; Gulbransen and Sharkey, 2012). Despite the realization that the different subpopulations of EGCs make critical and unique contributions to intestinal homeostasis, the dynamic relationship between spatially segregated EGCs, the physiological signals that regulate their steady-state equilibrium, and their response to trauma or disease remain unknown.

One of the subpopulations of EGCs that has generated considerable interest recently is located within the intestinal mucosa (Gulbransen and Sharkey, 2012; Rühl, 2005). In addition to their neuroprotective function, these mucosal EGCs (mEGCs) are thought to play crucial roles in maintaining the intestinal epithelial barrier and regulating immune responses in the mucosa (Bush et al., 1998; Neunlist et al., 2013; Rühl et al., 2004; Savidge et al., 2007). The residence of mEGCs within the most dynamic layer of the gut wall and their interactions with highly regenerative and remodeling tissues, such as the intestinal epithelium and the mucosal immune system, raise interesting questions regarding their development and homeostasis. These questions acquire renewed urgency given the emerging effects of microbiota on the organization and function of multiple GI tissues.

Here we have examined the developmental profile of mEGCs and their maintenance in adult mice. Our analysis shows that, in contrast to neural projections, mEGCs colonize the intestinal mucosa after birth. By performing inducible lineage tracing experiments we demonstrate that the network of mEGCs is maintained throughout life by the continuous supply of new glial cells originating in the peripheral plexi. Finally, by analyzing germ-free (GF), conventionalized, and antibiotic-treated mice we provide evidence that the postnatal settlement of mEGCs in the intestinal mucosa and the ongoing supply of glial cells to the lamina propria in adult mice are regulated by the gut microbiota. Our work provides insight into the role of environmental factors in the development of glial cells and their homeostasis in adult animals.

## Results

### The Network of mEGCs Develops after Birth

**Figure 1:**
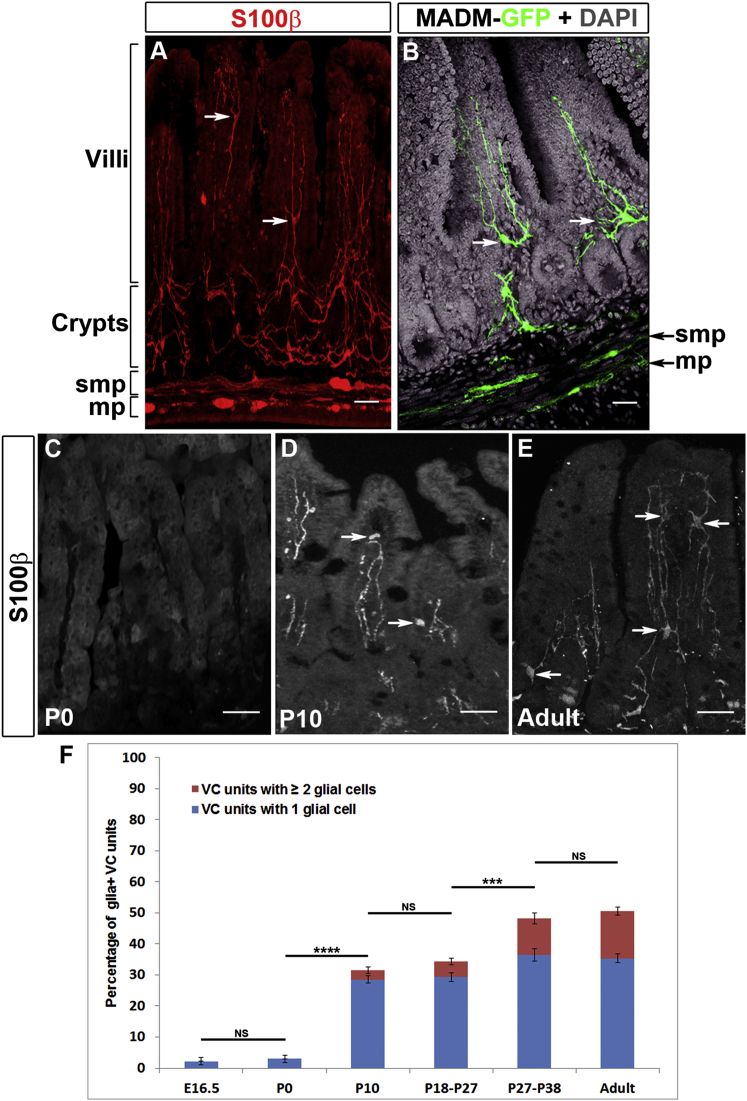
The Network of mEGCs Develops after Birth (A) S100β immunostaining of a vibratome cross-section from the ileum of an adult wild-type mouse. In addition to the myenteric (mp) and submucosal plexus (smp), EGCs (arrows) are also found within the lamina propria around the crypts and within villi. (B) Highly branched GFP^+^ glial cells (arrows) within the mucosa in the ileum of *Sox10::Cre;MADM*^*GR/RG*^ mice. (C–E) S100β immunostaining of cryosections of the mucosa of P0 (C), P10 (D), and adult (E) mice. Arrows in (D) and (E) point to mEGCs. (F) Quantification of glia^+^ VC units (GFP and S100β antibodies on sections from *Sox10::Cre;R26REYFP* mice) demonstrating that the network of mEGCs develops postnatally. Data are represented as mean of all glia+ VC units ± SEM. One-way ANOVA, p value < 0.0001: Tukey post hoc test showed that comparison of E16.5 to P0, P10 to P18–P27, and P27–P38 to Adult was not significant (NS). However, comparison of E16.5 to P10, P18–P27, P27–P38, and Adult; P0 to P10, P18–P27, P27–P38, and Adult; P10 to P27–P38 and Adult; and P18–P27 to Adult was significant (^∗∗∗∗^p < 0.0001). Comparison of P18–P27 to P27–P38 was also significant (^∗∗∗^p = 0.0003). The F (DFn, DFd) value is 227.6 (5, 12). Scale bars: 100 μm (A and B); 50 μm (C–E).

Immunostaining of sections from adult mouse intestine for the glia-specific marker S100β displayed a dense network of EGCs extending from the MP and SMP to the lamina propria between crypts and within villi (Figure 1A). To characterize in detail the morphology of mEGCs, we combined the *Sox10::Cre* driver (Matsuoka et al., 2005) with the *MADM-6*^*GR*^ and *MADM-6*^*RG*^ alleles (Zong et al., 2005) in order to express green fluorescent protein (GFP) in subsets of peripheral glial cells (Boesmans et al., 2015). mEGCs were highly branched (Figure 1B; Movie S1) and contacted several mucosal tissues, including the epithelium, blood vessels, and neurites (Figure S1; Movies S2 and S3) (Bohórquez et al., 2014; Liu et al., 2013). The apparent interaction of mEGCs with highly regenerative and remodeling tissues of the mucosa prompted us to examine their own dynamic properties. For this, we first analyzed the developmental profile of mEGCs by immunostaining intestinal sections from *Sox10::Cre;R26REYFP* reporter mice at different embryonic and postnatal stages with combinations of antibodies for S100β, YFP, and the neuronal marker PGP9.5. Neuronal fibers emanating from enteric ganglia were observed in ∼50% of villi at embryonic stage (E) 16.5 and in the majority of villi at postnatal day (P) 0 (Figure S2). In contrast, the lamina propria along the villus-crypt (VC) units (Figure S2) was essentially devoid of glial cells at both E16.5 and P0 (n = 3; Figures 1C and 1F). The small fraction of glia^+^ VC units identified at these stages contained a single glial cell (Figure 1F). At P10 (n = 3) the percentage of glia^+^ VC units increased significantly and units with more than one glial cell could also be identified (Figures 1D and 1F). These parameters increased further in adult (P ≥ 60, n = 3) mice (Figures 1E and 1F). To examine the potential role of weaning on the maturation of the mEGC network, we quantified mEGCs in the ileum of mice whose age ranged from P18 to P38. At the time of gut harvesting, three animals (P18, P21, and P27) were still at the home cage with their mother (pre-weaning group), while the remaining (P27, P32, and P38) had been weaned 7 days earlier (post-weaning group). The average values of the pre-weaning and post-weaning groups were significantly different but very similar to those from P10 and adult animals, respectively (Figure 1F). Moreover, comparison of the values of the two animals that belong to different groups but have same age (P27) suggests that weaning contributes to the expansion of mEGCs observed after P10. Together, our experiments suggest that the mEGC network develops in response to signals associated with adaptation of the GI tract to the postnatal environment of the lumen and nutrition.

### The Network of mEGCs Is Continuously Supplied with Glial Cells Originating in the Ganglionic Plexi

**Figure 2:**
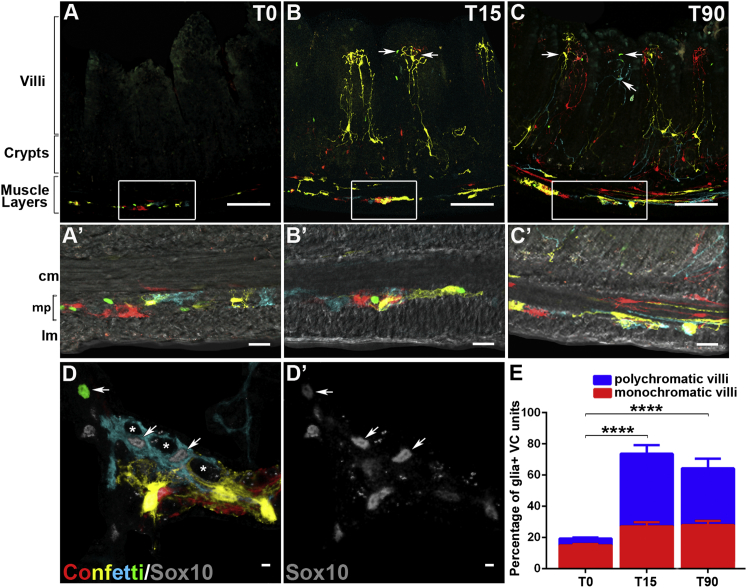
Inducible Lineage Tracing of mEGCs Distribution of confetti^+^ cells in the ileum of tamoxifen-treated *Sox10::CreER*^*T2*^*;R26RConfetti* mice at T0 (A), T15 (B), and T90 (C). Boxed areas in (A)–(C) correspond to panels (A′)–(C′), respectively. (D and D′) Confetti^+^ cells in a flat-mount preparation of myenteric ganglia from *Sox10::CreER*^*T2*^*;R26RConfetti* mice at T0. Arrows in (D) and (D′) indicate Sox10^+^Confetti^+^ glial cells. Asterisks in (D) indicate the position of confetti-negative enteric neurons. (E) Quantification of VC units with confetti^+^ glial cells at T0, T15, and T90. Data represented as mean ± SEM. Significant differences between the ages have been obtained with the two-way ANOVA, p < 0.0001, Tukey post hoc test, ^∗∗∗∗^p < 0.0001 (T0–T15;T0–T90), p = 0.7173 (T15–T90). Although VC units with monochromatic glia showed no significant change in number (p > 0.1), VC units with polychromatic glia showed significant differences: ^∗∗∗∗^p < 0.0001 (T0–T15; T0–T90), p = 0.6222 (T15–T90). The F(DFn, DFd) and p values for Factor 1: T0 Vs T15 Vs T90 is 29.94 (2, 18) with p = 0.08; Factor 2: polychromatic Vs monochromatic is 3.36 (1, 18) with ^∗∗∗∗^p < 0.0001 and interaction of Factor 1 with Factor 2 is 8.104 (2, 18) with ^∗∗^p = 0.0031. cm, circular muscle layer; lm, longitudinal muscle layer; mp, myenteric plexus. Scale bars: 100 μm (A–C); 50 μm (A′–C′); 20 μm (D and D′).

Given the regenerative and remodeling capacity of the intestinal mucosa, we considered the possibility that mEGCs detected within the lamina propria at any given moment represent a snapshot in the life cycle of a highly dynamic and incessantly renewing neuroectodermal cell population. Moreover, since ganglionic EGCs are capable of responding to injury and other insults (Joseph et al., 2011; Laranjeira et al., 2011), we posited that the ganglionic plexi of the outer gut wall constitute the main source of incoming mEGCs in adult animals. To test this hypothesis directly, we performed inducible lineage tracing of EGCs in adult mice by combining the *Sox10::CreER*^*T2*^ transgene, which drives expression of tamoxifen-inducible Cre recombinase in peripheral glial cells (Laranjeira et al., 2011), with the Cre-dependent multicolor *R26R-Confetti* reporter (Snippert et al., 2010). Transgenic mice 8–12 weeks old (n = 4) were administered tamoxifen and analyzed 4 days later (T0) to establish the baseline labeling pattern, and 15 or 90 days post-induction (T15 and T90, respectively; n = 4 for each time point) to follow the fate of labeled cells. As expected, at T0 confetti^+^ glial cells were identified within the MP and (to a lesser extent) the SMP (Figures 2A, 2A′, 2D, and 2D′). However, at this stage only 18% of VC units identified on sections contained labeled glial cells and the majority of them were monochromatic displaying a single confetti^+^ glial cell (Figures 2A and 2E). At T15 and T90, the number of confetti^+^ cells in the plexus was similar (or sometimes even increased), suggesting that the population of glial cells labeled at T0 remains stable at least during the post-induction period analyzed (Figures 2B, 2B′, 2C, and 2C′). Interestingly, at these stages numerous confetti^+^ EGCs were found in the majority of VC units (∼72% at T15 and ∼65% at T90) and were distributed along the entire crypt-villus axis (Figures 2B, 2B′ 2C, 2C′, and 2E). In contrast to T0, the majority of confetti^+^ VC units at T15 and T90 were polychromatic containing several glial cells labeled with different fluorescent reporters (Figures 2E and S3). The dramatic increase in the number of confetti^+^ cells within VC units and villi and their monochromatic to polychromatic switch at T15 and T90 indicated that during the post-induction period they are colonized by multiple lineally unrelated mEGCs originating from a labeled pool of glial cells in the ganglionic plexi.

To provide further support for the centripetal flow of glial cells along the serosa-lumen axis in adult mice under physiological conditions, we combined the *hGFAP::CreER*^*T2*^ transgene, which marks a relatively small subset of EGCs (Laranjeira et al., 2011), with the *R26RConfetti* and *R26REYFP* reporters. Three to four days after tamoxifen administration (T0) to 8- to 12-week-old mice from both driver-reporter combinations, sparsely labeled glial cells were detected predominantly in the MP (Figure S3). At this stage, no labeled glial cells were identified in the lamina propria (0/169 YFP^+^ cells detected on sections from the ileum of *hGFAP::CreER*^*T2*^*;R26REYFP* mice; n = 4). In contrast, 8–12 weeks post-induction ∼9.5% of YFP^+^ cells were located within the mucosa (Figure S3; 24/253 YFP^+^ cells identified; n = 4) with the remaining residing within the ganglia. Taken together, our in vivo lineage tracing experiments demonstrate that in adult mice the pool of mEGCs is continuously supplied by new cells originating from Sox10^+^ and GFAP^+^ glia located within the peripheral ganglia.

### Microbiota Is Required for the Postnatal Development of mEGCs

**Figure 3:**
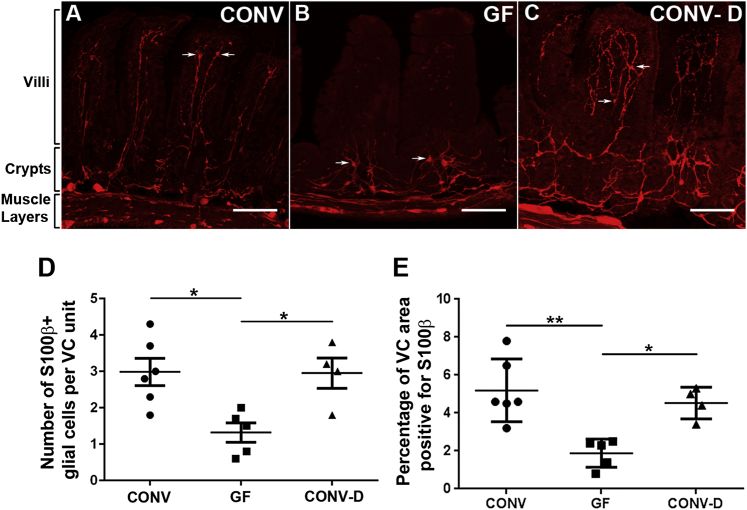
Formation of the mEGC Network Depends on Microbiota Cross-sections from the ileum of CONV (A), GF (B), and CONV-D (C) mice immunostained for S100β. Arrows indicate glial cells. (D) Quantification of S100β^+^ cells in the three conditions. One-way ANOVA, p value = 0.0093, Tukey post hoc test, ^∗^p = 0.0128 (CONV versus GF), ^∗^p = 0.0260 (GF versus CONV-D). Glial populations were not significantly different between CONV and CONV-D mice. The F(DFn, DFd) value is 7.086 (2, 12). (E) Percentage of VC area positive for S100β is represented in all three conditions. One-way ANOVA, p value < 0.05, Tukey post hoc test, ^∗∗^p = 0.002 (CONV versus GF), ^∗^p = 0.018 (GF versus CONV-D). The F(DFn, DFd) value is 10.70 (2, 12). Note that the glial cell number and distribution and the area of VC units positive for S100β was similar between CONV and CONV-D animals, but significantly different from GF mice. Scale bars: 100 μm (A–C). CONV = conventional; GF = germ-free; CONV-D = conventionalized.

Recent work has demonstrated that the gut microbiota influences the function of neuronal circuits (Diaz Heijtz et al., 2011), but much less is known about the communication between the ENS and intestinal microflora. The GI tract is colonized immediately after birth with micro-organisms that increase in number and complexity after weaning (Nicholson et al., 2012). Since the postnatal development of mEGCs coincides with the establishment and maturation of microbiota, we examined the potential link between the two processes by comparing the mEGC network in the ileum of 8-week-old conventionally raised (CONV; n = 6) and germ-free (GF; n = 5) mice (Figure S4). Interestingly, although no obvious difference in S100β immunostaining was observed in the MP and SMP between the two groups, the average number and density of mEGCs in the mucosa of GF mice was significantly reduced relative to CONV animals (Figures 3A, 3B, 3D, and 3E). The reduction of mEGCs in GF mice was not uniform along the crypt-villus axis since mEGCS were essentially eliminated from the villi but those at the level of the crypts were less affected (Figures 3A and 3B). To examine whether the requirement of the gut microbiota for normal EGC development is restricted to a critical early postnatal period, 4-week-old GF mice were conventionalized (CONV-D) by gavage feeding of microbiota and subsequent co-housing with CONV animals (Figure S4) and intestinal sections were immunostained for S100β 4 weeks later. In comparison to GF mice, the average number of mEGCs in the mucosa of CONV-D animals (n = 4) was increased and the network of glial cells within the lamina propria of villi was restored (Figures 3B–3E). We conclude that normal development of the mEGC network depends on gut microbiota but the ability of EGCs to invade the intestinal mucosa is not restricted to the early postnatal life.

### Microbiota Regulates the Continuous Centripetal Flow of mEGCs along the Serosa-Lumen Axis

**Figure 4:**
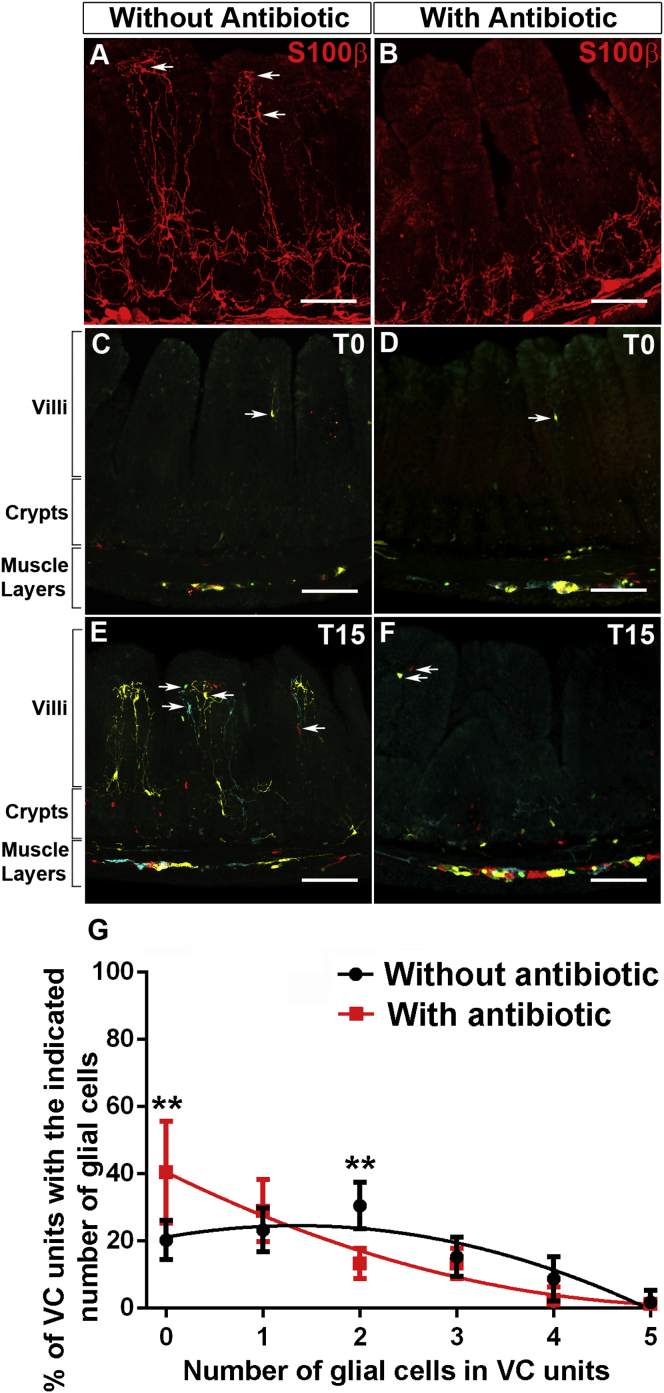
Microbiota Controls the Continuous Supply of Glial Cells to the Intestinal Mucosa (A and B) Sections of the ileum of control (A) and antibiotic-treated (B) wild-type adult mice. Arrows in (A) indicate glial cells. (C–F) Distribution of confetti^+^ glial cells in the ileum of tamoxifen-treated 8- to 12-week-old *Sox10::CreER*^*T2*^*;R26RConfetti* mice at T0 (C and D) and T15 (E and F) in the absence (C and E) or presence (D and F) of antibiotics. Arrows indicate confetti^+^ glial cells in the lamina propria. (G) The average number of confetti^+^ cells in VC units at T15 is reduced in antibiotic-treated animals. Data is represented as mean ± SEM. The distribution of the number of labeled glial cells per VC unit has been plotted using the non-linear paradigm, representing a different curve for each dataset as the best fit with a ^∗∗∗^p value = 0.0004. Using the two-way ANOVA and Sidak’s multiple comparisons test, significant differences were observed in the 0 and 2 cells per VC unit categories with ^∗∗^p values = 0.0014 and 0.0081, respectively. The F(DFn, DFd) and p values for Factor 1: With Vs Without antibiotic is 0 (1, 36) p > 0.99; Factor 2: number of glial cells in VC units is 21.47 (5, 36) ^∗∗∗∗^p < 0.0001 and interaction of Factor 1 with Factor 2 is 6.353 (5, 36) with ^∗∗∗^p = 0.0003. Scale bars: 100 μm (A–F).

To examine whether gut microbiota is required for maintaining the normal complement of mEGCs throughout life, 8- to 12-week-old mice were treated with antibiotics for 3 weeks and the ileum was immunostained for S100β. Antibiotic treatment reduced the number of micro-organisms within the gut lumen and led to cecum enlargement (Reikvam et al., 2011) (Table S1; Figure S4). Importantly, it also reduced the number of S100β^+^ glial cells within the mucosa (Figures 4A and 4B) with mEGCs within villi affected more relative to their counterparts around the crypts (Figures 4A and 4B). Analysis of gut sections from control and antibiotic-treated *Sox10::Cre;R26RConfetti* mice, in which all mEGCs were labeled by confetti-encoded fluorescent reporters, confirmed that antibiotic treatment reduced the number of mEGCs rather than the expression levels of the S100β marker (Figure S4). Finally, to examine whether antibiotics disrupt the homeostatic flow of EGCs along the plexus-crypt-villus axis, we combined antibiotic treatment and EGC lineage tracing in 8- to 10-week-old *Sox10::CreER*^*T2*^*;R26RConfetti* mice. Administration of antibiotics resulted in dramatic reduction of the polychromatic mEGC population observed in the mucosa two weeks after tamoxifen administration (Figures 4C–4G). Taken together, these experiments argue that signals emanating from gut microbiota initiate and sustain the flow of mEGCs from the peripheral plexi to the lamina propria.

## Discussion

Despite extensive literature on the self-renewal and remodeling of non-neuroectodermal intestinal tissues (Belkaid and Hand, 2014; Clevers, 2013; Stappenbeck et al., 2002), only limited information is available on the homeostasis of the intrinsic neuroglial networks of the gut wall. Indeed, the ENS is often portrayed as an assembly of ganglionic units that form during development and are maintained, perhaps unchanged, throughout life. Here we challenge this view and demonstrate that the ostensibly rigid neuroglial networks in the gut of adult animals encompass highly dynamic populations of EGCs with previously unrecognized hierarchical relationships. The extensive network of mEGCs, which develops postnatally over a period entailing dramatic changes in GI microenvironment and physiology, continues to be renewed in adult animals by incoming glial cells originating in the ganglionic plexi of the gut wall. We also demonstrate that both the initial establishment and the homeostasis of mEGCs are controlled by the gut microbiota (Figure S4).

In our lineage tracing studies, the supply of new mEGCs to the intestinal mucosa was not associated with reduction of the labeled cell population in MP and SMP, suggesting that the subset of glial cells targeted by our reporter system is long lived. It would be interesting to determine whether the continuous centripetal flow of mEGCs is supported by dedicated progenitors or differentiated glial cells, which under certain conditions acquire self-renewing potential. Irrespective of the identity of mEGC progenitors in the ganglionic plexi, our current observations provide a rationale for the constitutive gliogenesis observed in the MP of adult rodents (Joseph et al., 2011; Laranjeira et al., 2011). Consistent with our current studies and in further support of the dynamic state of EGCs in the adult gut, pharmacogenetic ablation of proliferating glial cells in mice led to epithelial barrier breakdown and inflammatory degeneration of the mucosa (Bush et al., 1998; Savidge et al., 2007).

Although the majority of neurons found in MP and SMP of adult mice are already in place by the end of fetal life, enteric neurogenesis continues for several weeks after birth (Laranjeira et al., 2011) extending beyond the early postnatal period of microbiotal establishment and maturation. Interestingly, GF rodents are characterized by reduction in specific subpopulation of enteric neurons and abnormal peristalsis, suggesting that microbiota influence the development and maturation of intestinal neural networks (Abrams and Bishop, 1967; Collins et al., 2014; McVey Neufeld et al., 2013). Although the mechanisms by which luminal microflora impact the organization and function of enteric neural circuits remain largely unexplored, a recent study provided evidence for a reciprocal interaction between macrophages of the muscularis externa and enteric neurons that can be influenced by microbiota (Muller et al., 2014). The identification of EGCs as a major cellular target of intestinal microbiota in our study, in conjunction with the widely established roles of these cells in the survival and functional connectivity of enteric neurons, suggests that at least some of the motility defects observed in GF and antibiotic-treated rodents could be secondary to deficits in glial cell dynamics. For example, reduction of mEGCs that are associated with the projections of afferent neurons in the mucosa may result in altered input to the reflex circuitry that controls the pattern and frequency of peristaltic contractions of the gut wall.

The altered organization and properties of EGCs in GF rodents may also influence GI function independently of their effects on neural activity. Of particular interest in this context are the described roles of mEGCs in epithelial barrier function and immune responses in the intestinal mucosa (Bush et al., 1998; Neunlist et al., 2013; Rühl et al., 2004; Savidge et al., 2007). The implication of mEGCs in the function of multiple gut tissues, in conjunction with our current findings, suggests that the ongoing supply and turnover of glial cells to the lamina propria constitutes a novel microbiota-driven homeostatic mechanism essential for maintaining GI function under physiological conditions. It would be interesting to examine the dynamics of mEGCs in common inflammatory conditions of the gut (such as Crohn’s disease) and whether altered homeostasis of these cells could be implicated in the pathogenesis of certain functional GI disorders (including irritable bowel syndrome) that are associated with abnormal motility (Longstreth et al., 2006) and changes in the microbial environment (De Palma et al., 2014; Mayer et al., 2014; Simrén et al., 2013). In that respect it is interesting that in our studies the network of mEGCs was restored within the intestinal mucosa upon conventionalization of GF mice at a stage beyond an early critical postnatal period. Although it is currently unclear whether this apparent plasticity of EGCs is manifested at all stages of adult life, it is conceivable that restoring glial function in patients with dysbiosis (abnormal composition of microbiome) could alleviate at least some of the dysmotility-associated symptoms.

As the ENS (and EGCs in particular) are known to express microbial pattern recognition receptors (such as TLR2 and TLR4) (Barajon et al., 2009; Brun et al., 2013), it will be of interest to determine whether microbiota control the dynamics of enteric glia directly or via an intermediary cell type. Irrespective of the mechanisms, our experiments identify EGCs as a major target of gut microbiota and suggest that microbes and their products regulate the development and maturation of other glial cell networks of the nervous system. Consistent with this view, previous studies have demonstrated transcriptome changes of differentiated astrocytes and reprogramming of Schwann cells exposed to lipopolysaccharides and leprosy bacilli, respectively (Hamby et al., 2012; Masaki et al., 2013). Changes in the organization and function of enteric glia, an essential component of a key relay station (ENS) along the gut-brain axis, or potential deficits of CNS glia, could contribute to neuroendocrine and behavior abnormalities associated with changes in gut microbiota. Although our studies thus far have focused on the distal ileum, the identification of mEGCs in all intestinal segments, including the duodenum and the colon, suggests that homeostatic regulation of mucosal glia is a feature of the entire intestine. It will be interesting to determine whether microbiota regulates the homeostasis of mEGCs in all segments of the mammalian intestine and identify additional factors that contribute to the generation and supply of enteric glial cells to the intestinal mucosa. Understanding further the interplay between microflora and EGCs will provide an excellent model system to examine the effects of commensal and pathogenic micro-organisms on the host nervous system and help elucidate the pathogenesis and ultimately develop novel therapeutic strategies for GI disorders.

## Experimental Procedures

Experimental Procedures are described in Supplemental Information.

## Supplemental Information

Document S1. Figures S1–S4, Table S1, and Supplemental Experimental Procedures

Movie S1, Related to Figure 1. mEGCs Are Highly Branched Shown is a surface-rendered reconstruction of confocal images of a single GFP^+^ glial cell (green) in a villus of *Sox10::Cre;MADM*^*GR/RG*^ mice.

Movie S2, Related to Figure 1. mEGCs Are Closely Associated with Crypts Shown is a surface-rendered reconstruction of confocal images of crypt epithelial cells immunostained for A33 (cyan) in close association with GFP^+^ glial cell network (green) from the ileum of a *Sox10::Cre;MADM*^*GR/RG*^ mouse. Parts of the vascular network are highlighted by Rhodamine dextran sulphate (red).

Movie S3, Related to Figure 1. mEGCs Interact Closely with the Vascular Network of Villi Shown is a surface-rendered reconstruction of confocal images of a GFP^+^ glial cell (green) and Rhodamine Dextran-labeled microvasculature (red) in a villus of a *Sox10::Cre;MADM*^*GR/RG*^ mouse.

Document S2. Article plus Supplemental Information
