# Supplementary material for: Microbiota Controls the Homeostasis of Glial Cells in the Gut Lamina Propria
Source: Neuron. 2015 Jan 21;85(2):289–95. doi: 10.1016/j.neuron.2014.12.037 (PMC4306542; doi:10.1016/j.neuron.2014.12.037)
Supplement: Document S1. Figures S1–S4, Table S1, and Supplemental Experimental Procedures [file mmc1.pdf]

Neuron

Supplemental Information

Microbiota Controls the Homeostasis of Glial Cells in the Gut Lamina Propria

Panagiotis S. Kabouridis, Reena Lasrado, Sarah McCallum, Song Hui Chng, Hugo J. Snippert, Hans Clevers, Sven Pettersson, and Vassilis Pachnis

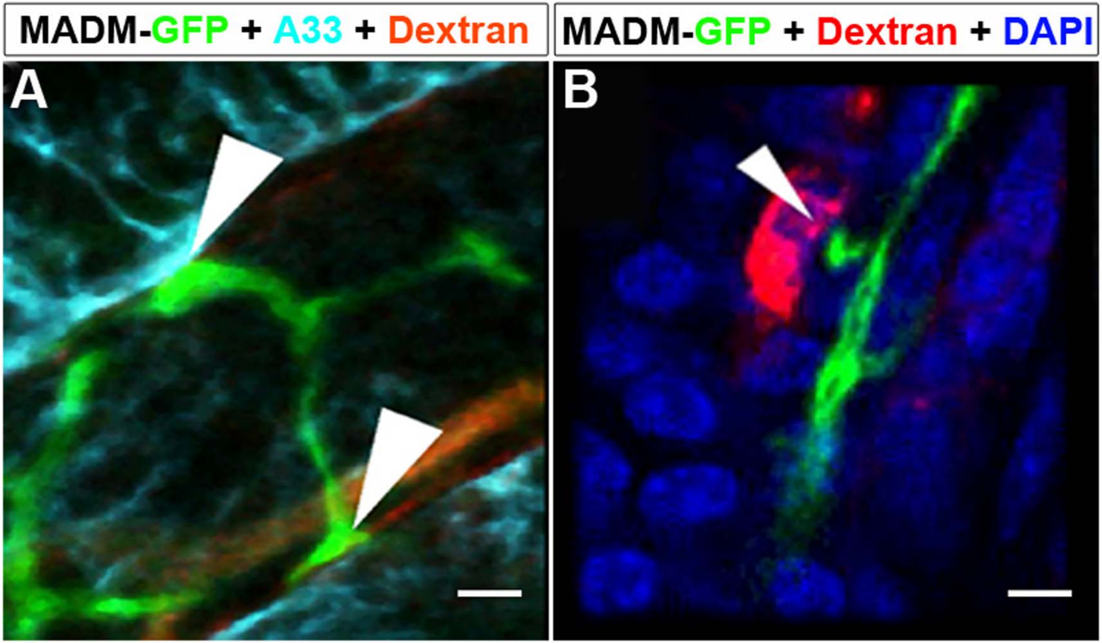

Kabouridis et al. Fig. S1 related to Fig. 1

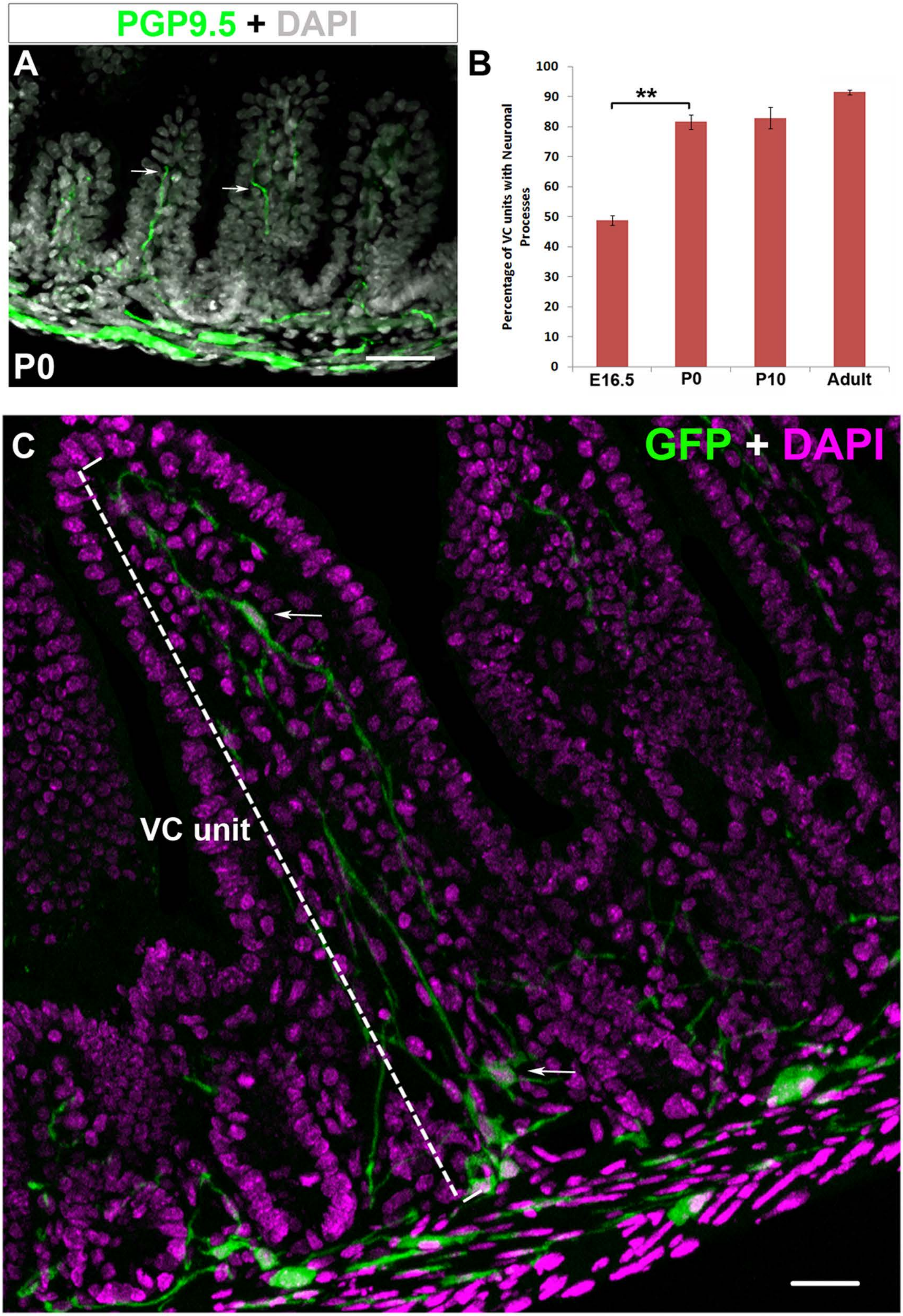

Kabouridis et al. Fig. S2 related to Fig. 1

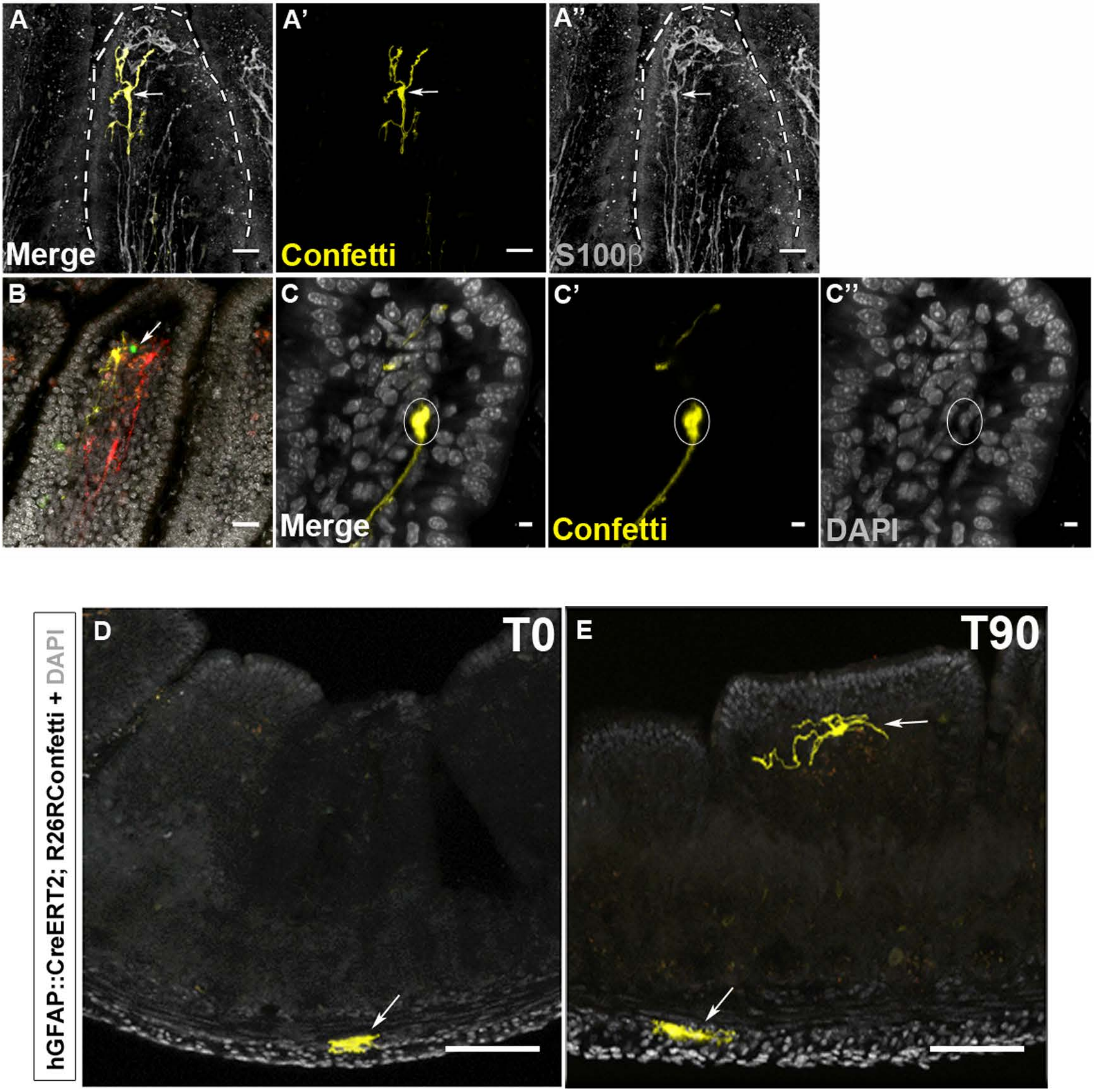

Kabouridis et al. Fig. S3 related to Fig.2

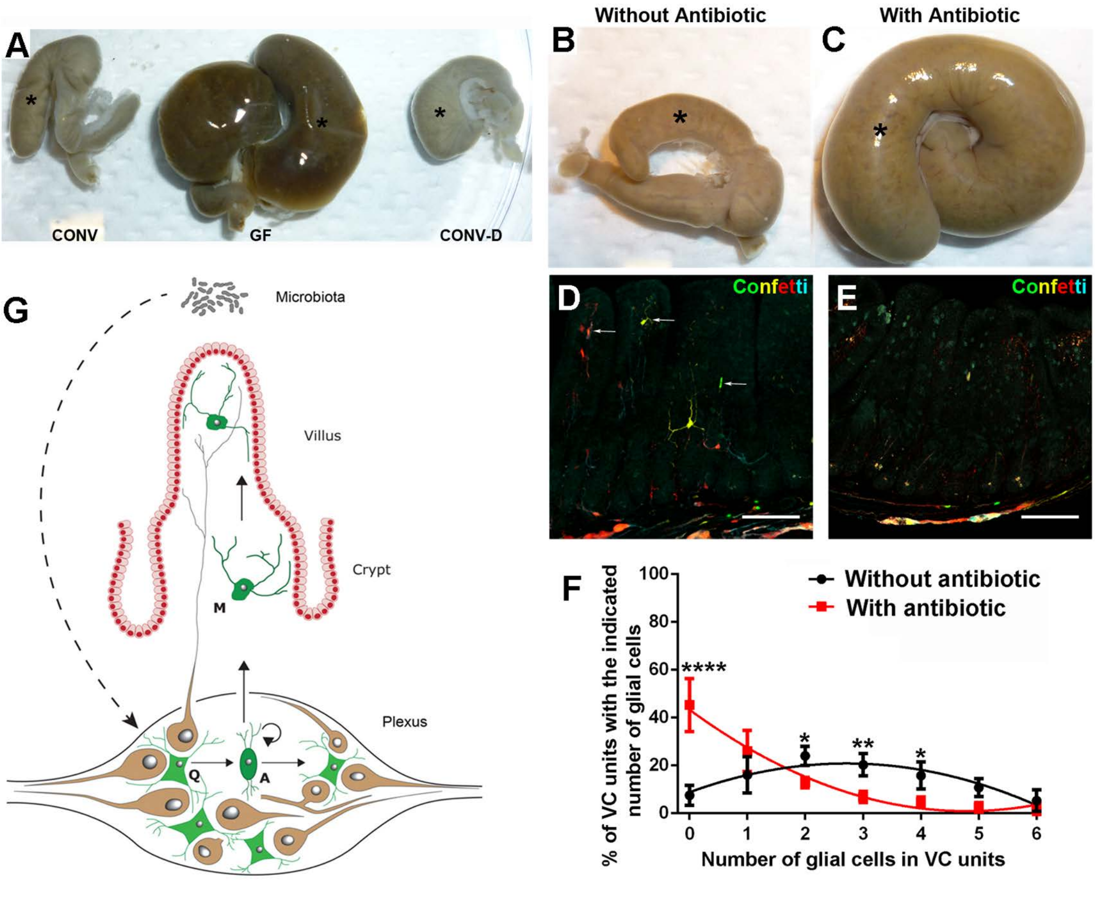

Kabouridis et al. Fig. S4 related to Fig. 3 and Fig. 4

SUPPLEMENTAL FIGURE LEGENDS

Figure S1, related to Figure 1. mEGCs contact multiple tissues in the intestinal mucosa.

(**A, B**) Glial processes (green, indicated by arrowheads) in the mucosa of *Sox10::Cre;MADM*$^{GR/RG}$ mice are juxtaposed to crypt epithelia (identified by A33 immunostaining; cyan) (**A**) and the vascular network (identified by Rhodamine Dextran; red) (**B**). Scale bars: 0.5μm.

Figure S2, related to Figure 1. Intestinal mucosa is innervated prior to colonization by mEGCs.

(**A, B**) Most villi of *Sox10::Cre;R26REYFP* P0 mice have been colonised by neuronal processes (green). Arrows in (**A**) indicated PGP9.5$^+$ neuronal processes. Scale bar: 100μm. (**B**) Quantification of villi with identifiable neuronal processes in the mucosa of *Sox10::Cre;R26REYFP* mice at different developmental stages. One way ANOVA, $P<0.05$, Tukey post-hoc test, was performed. The F (dFn, dFd) and p value is 18.12 (3, 6) with **p=0.002. (**C**) A villus-crypt (VC) unit corresponds to the lamina propria within a clearly identifiable villus and the associated crypt area above the submucosal plexus. Image is from a cryosection of the ileum of an adult *Sox10::Cre;R26REYFP* mouse. Arrows point to mEGCs identified DAPI$^+$ nuclei and the associated GFP$^+$ cytoplasm. Scale bar: 50μm.

Figure S3, related to Figure 2. Enteric glial cells continuously colonize the lamina propria of the gut.

(**A, A', A''**) A YFP$^+$ cell (indicated by arrow) in a villus from the ileum of a tamoxifen-treated *Sox10::CreER*^{T2}*;R26RConfetti* mouse at T15 co-expresses S100β. (**B**) A polychromatic villus from the ileum of a tamoxifen-treated *Sox10::CreER*^{T2}*;R26RConfetti* mouse at T15. The presence of GFP$^+$ nuclei (arrow) among the other fluorescent-labelled cells indicates that confetti signal corresponds to glial cell processes as well as glial cell bodies. This is independently confirmed by the superimposition of DAPI$^+$ nuclei and confetti signal (**C, C', C''**). Scale bars: 50μm (A-B), 25μm (C-C''). (**D, E**) Cross sections from the gut of adult *hGFAP::CreER*^{T2}*;R26RConfetti* mice injected with a single dose of tamoxifen and analysed at T0 (**D**) or at T90 (**E**). Arrows indicate isolated YFP$^+$ cells in the myenteric plexus (**D**) and the myenteric plexus and a villus (**E**). Scale bars: 100μm (D-E).

Figure S4, related to Figure 3 and Figure 4. Microbiota is required to maintain the normal complement of mEGCs in the gut lamina propria.

(A) Direct comparison of the size of ceca (asterisks) dissected from CONV (conventional), GF (germ-free) and CONV-D (conventionalized) mice. (**B-E**) Treatment of adult *Sox10::Cre;R26RConfetti* mice with broad spectrum antibiotics result in enlargement of cecum (**B, C**) and loss of Confetti$^+$ mEGCs (arrows) from the mucosa (**D, E**). Note the presence of Confetti$^+$ ganglia in the myenteric and submucosal plexi of antibiotic treated mice (**E**). (**F**) The average number of confetti$^+$ cells in VC units at T15 is reduced in antibiotic-treated animals. Data is represented as mean ± SEM. The distribution of the number of labelled glial cells per VC unit has been plotted using the non-linear paradigm, representing a different curve for each data set as the best fit with p value < 0.0001. Using two-way ANOVA and Sidak's post-hoc test significant differences were observed in the 0 (****p<0.0001); 2 (*p=0.03); 3 (**p=0.0067) and 4 (*p=0.04) cells per VC unit categories. The F(DFn, DFd) and p values for Factor 1: With Vs Without antibiotic is 0 (1, 42) p>0.98; Factor 2: number of glial cells in VC units is 18.43 (6, 42) ****p<0.0001 and interaction of Factor 1 with Factor 2 is 23.46 (6, 42) with ****p<0.0001. Asterisks in panels **A** and **B** indicate the ceca. Scale bars in D-E: 100µm. (**G**) Model presenting the regulation of the homeostatic flow of glial cells from the peripheral plexus to the lamina propria of the gut mucosa. The microbiota of the gut lumen, either directly or indirectly, promote the transition of mature quiescent (Q) glial cells within the myenteric or submucosal plexi to an active (A) state which gives rise to mEGC (M) progeny that migrate into the mucosa. This model is consistent with the low levels of EGC proliferation and constitutive enteric gliogenesis that has been observed in the gut of adult rodents (Joseph et al., 2011; Laranjeira et al., 2011).

SUPPLEMENTAL TABLE

Table S1

**Mice:**<br>**Microorganisms:**	**With antibiotics**	**Without antibiotics**
**Aerobic bacteria**		
0 days	4.3×10^6 ($\pm 3.2 \times 10^6$)	4.9×10^6 ($\pm 2.6 \times 10^6$)
10 days	<1	1.0×10^6 ($\pm 0.34 \times 10^6$)
22 days	<1	6.6×10^5 ($\pm 2.8 \times 10^5$)
**Anaerobic bacteria**		
0 days	29.8×10^6 ($\pm 31 \times 10^6$)	8.2×10^6 ($\pm 4.3 \times 10^6$)
10 days	<1	4.2×10^6 ($\pm 5.0 \times 10^6$)
22 days	<1	1.5×10^6 ($\pm 1.16 \times 10^6$)
**Yeast**		
0 days	1.66×10^5 ($\pm 0.12 \times 10^5$)	1.5×10^5 ($\pm 0.65 \times 10^5$)
10 days	<1	2.0×10^4 ($\pm 0.65 \times 10^5$)
22 days	<1	1.0×10^4 ($\pm 1.1 \times 10^4$)

Table S1, related to Figure 4. Bacteriologic analysis of faecal pellets in control and antibiotic-treated mice. Antibiotic treatment for 10 days reduced dramatically the number of aerobic and anaerobic bacteria and yeasts. Similar reduction was also observed at the end of the 3-week antibiotic course.

SUPPLEMENTAL MOVIES

Movie S1, related to Figure 1. mEGCs are highly branched. Shown is a surface-rendered reconstruction of confocal images of a single GFP^+ glial cell (green) in a villus of *Sox10::Cre;MADM*$^{GR/RG}$ mice.

Movie S2, related to Figure 1. mEGCs are closely associated with crypts. Shown is a surface-rendered reconstruction of confocal images of crypt epithelial cells immunostained for A33 (cyan) in close association with GFP^+ glial cell network (green) from the ileum of a *Sox10::Cre;MADM*$^{GR/RG}$ mouse. Parts of the vascular network are highlighted by Rhodamine dextran sulphate (red).

Movie S3, related to Figure 1. mEGCs interact closely with the vascular network of villi. Shown is a surface-rendered reconstruction of confocal images of a GFP^+ glial cell (green) and Rhodamine Dextran labelled microvasculature (red) in a villus of a *Sox10::Cre;MADM*$^{GR/RG}$ mouse.

MATERIALS AND METHODS

Animals

Generation of the *R26REYFP* (Srinivas et al., 2001), *R26RConfetti* (Snippert et al., 2010), *MADM-6*^{GR} and *MADM-6*^{RG} reporter (Tasic et al., 2012; Zong et al., 2005) and the S*ox10::CreER*^{T2} (Laranjeira et al., 2011), *hGFAP::CreER*^{T2} (Ganat et al., 2006) and *Sox10::Cre* (Matsuoka et al., 2005) transgenic mice have been described previously. The effect of microbiota on mEGCs was examined in wild-type C57Bl/6 mice which were raised either conventionally at specific pathogen-free conditions (CONV animals) or in germ-free isolators (GF animals). Sterility of isolators was analysed weekly by plating fecal homogenates onto different types of agar plates to detect aerobic and anaerobic bacteria and fungi. Mice maintained in GF conditions were given autoclaved food (LabDiet 5010/5021) and drinking water. Consistent with previous reports (Reinhardt et al., 2012), we consistently observed that the villi of GF mice were generally thinner relative to those from CONV animals. GF mice were conventionalised (CONV-D animals) by gavaging 100µl of homogenate generated by dissolving 2 fecal pellets from CONV animals in 1 ml of PBS. Following gavage CONV-D mice were co-housed with CONV animals. All mice were maintained in the animal facilities of NIMR (London, UK) and SingHealth Research (Singapore) in accordance with the regulatory standards of each institution and experiments were approved by the respective local ethical committees.

Tamoxifen administration for lineage tracing of EGCs

4-OH-tamoxifen was prepared at stock concentration of 10 mg/ml in corn oil with 10% ethanol. 8-12 week old *Sox10::CreER*^{T2}*;R26RConfetti* mice were administered 4-OH-Tamoxifen (0.1 mg/gram body weight) intraperitoneally for two consecutive days and analysed 4 days (T0), two weeks (T15) or three months (T90) later without additional exposure to tamoxifen. *hGFAP::CreER*^{T2}*;R26RYFP* mice were induced with a single injection of 4-OH-Tamoxifen (0.1 mg/gram body weight) and analysed at the indicated stages.

Antibiotic-induced depletion of gut microbiota

The effect of microbiota on the maintenance of mEGCs was analysed in 8-12 week-old wild-type (Parkes) and *Sox10::Cre;R26RConfetti* reporter mice that were supplied with a cocktail of broad-spectrum antibiotics in their drinking water for 3 weeks. The antibiotic cocktail consisted of ampicillin (1gr/L; Centaur Services Ltd.), metronidazole (1 gr/L; Centaur Services Ltd.), vancomycin (0.5 gr/L; Centaur Services Ltd.) and neomycin (0.5 gr/L; Sigma-Aldrich) (Reikvam et al., 2011). Drinking water was sweetened with 1% w/v of sucrose and

fresh antibiotic preparation was administered every 4 days. For the combination of EGC lineage tracing and antibiotic treatment, 8-12 week-old *Sox10::CreER*^{T2}*;R26RConfetti* mice were induced with 4-OH-tamoxifen one week following addition of antibiotics to the drinking water and the distribution of labelled glial cells was analysed at T0 and T15. The effect of antibiotics on intestinal microflora was examined by quantitative analysis of aerobic and anaerobic bacteria and yeasts present in faecal pellets of *Sox10::Cre;R26RConfetti* mice. On day 0 of antibiotic treatment (prior to the addition of the antibiotic cocktail to the drinking water), on day 10 and on day 22 (conclusion of the treatment), mice defecated directly into pre-weighted sterile microcentrifuge tubes. Pellets were dissolved in PBS and 100μl of serial dilutions of faecal suspensions were plated onto duplicate horse blood agar plates for aerobic incubation (with 5% CO_2), anaerobic horse blood agar plates (incubation in anaerobic chamber) and Saboraud Dextrose agar for yeast counts (air incubation). Following 3 days of incubation, plates were enumerated and the number of micro-organisms in the original sample was calculated and expressed as colony-forming units per milligram of faecal material (cfu/mg). Faecal bacterial load was determined for three mice in each of the antibiotic treatment and control groups (**Table S1**).

Marking the vascular network of MADM mice

To enable visualisation of the mucosal vascular system, *Sox10::Cre;MADM-6*$^{GR/RG}$ were placed briefly under a heat lamp to dilate the tail vein, which was used to inject of 50μl of 2000kDa Rhodamine Dextran (10mg/ml in PBS; Life Technologies). 5 min after injection animals were culled by cervical dislocation.

Developmental profile of mEGCs

The developmental profile of mEGCs was analysed in the ileum of *Sox10::Cre;Rosa26EYFP* mice in which all enteric neurons and glia are labelled by GFP (Laranjeira et al., 2011). Since in rodents the cell somata of enteric neurons are located exclusively within the ganglia of the myenteric and submucosal plexus, GFP$^+$ cells within the mucosa represent S100β$^+$ glial cells. Due to the extensive branching of glial cells, we used the robust cytoplasmic GFP signal in association with the nuclear marker DAPI to quantify the experiments shown in **Figure 1F**. Glial cells were quantified in the area of the lamina propria that corresponds to villus-crypt (VC) units. Each VC unit represents a clearly identifiable villus and the associated crypt area extending up to (but excluding) the submucosal and myenteric plexus (**Figure S2**). Quantification is based on analysis of at least 200 VC units from each animal and at least three animals were used for each stage. For all stages included in the developmental analysis, gut was dissected and fixed in 4% paraformaldehyde (PFA) at 4^0C overnight (O/N).

Following extensive washing with PBS 1cm segment of the ileum was placed in 30% sucrose (in PBS) and incubated O/N at 4^{0}C. Gut samples were embedded in a mix of 15% sucrose and 7.5% gelatin in PBS. When solidified at 4^{0}C, tissue blocks were immersed in isopentane on dry ice and stored at -80^{0}C until processed further. 14μm sections were generated using a Leica Cryostat and placed on Superfrost Plus slides. For immunostaining, sections were permeabilised with 0.3% PBT (PBS/TritonX-100) for 10 minutes and incubated with blocking solution (1% Bovine Serum Albumin and 0.15% Glycine in 0.1% PBT) for at least 1 hour. Combinations of primary antibodies were diluted as required in blocking solution and incubated O/N at 4^{0}C. Secondary antibodies were diluted in blocking solution and applied to sections at room temperature (RT) for 2 hours. Following three washes with 0.3% PBT, slides were mounted with mounting medium containing DAPI. Primary antibodies: S100β (rabbit polyclonal from DakoCytomation), PGP9.5 (rabbit polyclonal from Serotec) and GFP (rat monoclonal, clone GF090R, from Nacalai Tesque). All primary antibodies were used at a dilution of 1:1000. Secondary antibodies were AlexaFluor564-conjugated donkey anti-rabbit (Jackson ImmunoResearch) and AlexaFluor488-conjugated donkey anti-rat (Invitrogen) and used at a dilution of 1:500.

S100β immunostaining and fluorescent reporter detection in the gut of adult mice

Ileum from 8-12 week-old mice was immersed in 4% PFA in PBS for 4 hours at RT and fixed for a further of 16 hours in 2%PFA in PBS at 4^{0}C. Following fixation, tissue was washed extensively and either processed immediately or stored at 4^{0}C in PBS (with 0.05% sodium azide) for up to 1 week. 4-5mm long segments from the terminal ileum were embedded in 3% agarose in PBS and sectioned with a vibratome (Leica VT1000S) to obtain 50μm sections. For imaging of fluorescent reporters (MADM-GFP, Confetti) sections were mounted directly onto slides and analysed by laser confocal microscopy. Flat-mount preparations of myenteric ganglia were generated by peeling the longitudinal muscle layer of a freshly-dissected segment of the ileum along with the attached myenteric plexus (Laranjeira et al., 2011) followed by fixation for 30 min in 4% PFA in PBS. For immunostaining, sections or peels were blocked in 5% normal donkey serum in PBS/0.5% TritonX-100 for 2 hours at RT and incubated with primary antibodies diluted in blocking solution O/N. Following extensive washing sections or peels were incubated with secondary antibodies for at least 2 hours at RT. Primary antibodies: S100β (rabbit polyclonal from DakoCytomation, 1:1000), Sox10 (rabbit polyclonal from Santa Cruz, 1:200), and GFP (rat monoclonal, clone GF090R, from Nacalai Tesque, 1:1000). Secondary antibodies were AlexaFluor 564-

conjugated donkey anti-rabbit (Jackson ImmunoResearch, 1:500) and AlexaFluor 488-conjugated donkey anti-rat (Invitrogen, 1:500).

To assess the effect of GF conditions and antibiotics on mEGCs we examine >2000 VC units from each gut analysed. In virtually all cases, glial cells were eliminated from the villi but some S100β cells were often observed in the lamina propria between crypts (**Figure 3B and Figure 4B**). Since the number and position of the residual glial cells varied along the crypt-villus axis, the effect of GF conditions and antibiotic treatment was quantified by counting the number of S100β^{+} glial cells present along the entire length of ~100 randomly identified VC units. To determine the area of VC units occupied by glial cells images of S100β immunostainings were converted into binary images (8-bit grayscale) with ImageJ and the area of individual VC units was selected. The S100β area scoring above the set threshold was expressed as fraction of the total area selected using the Measure command under the Analyse tool. An average value of the VC unit measurements was calculated for every mouse in each group.

Confocal microscopy and image analysis

Confocal images of adult gut cryostat (14µm) and vibratome (50µm) sections were acquired with Leica TCS SP5 with a DN6000 microscope assisted by the LAS AF software. The objective lenses used included a dry X20 (0.75 NA), Nose-dip X20 (1.0 NA) and an oil-immersion X40 (1.75 NA). Sequential scans were performed in for XFP excitations: nuclearGFP, the argon laser 488 nm line; for EYFP 514 nm line; for RFP a red diode laser emitting at 561 nm, and blue mCFP was excited using a laserline at 458 nm. In general GFP fluorescence was collected between 498–510nm, airy 1; EYFP fluorescence was collected between 521–560 nm, airy 1; RFP fluorescence was collected between 590–650 nm, airy 1; mCFP fluorescence was collected between 466–495 nm, airy 1.5. DIC was obtained while using 488 nm laser through transmission gate. All images were processed with either Adobe Photoshop CS4 (Adobe Systems) or ImageJ (Wayne Rasband, NIH). 3-D reconstructions and movies were generated using the 3D opacity mode in Volocity (Perkin Elmer, Waltham, MA).

Quantification and Statistical Analysis

Data have been obtained from at least three independent animals. N values have been mentioned in the text/figure legends. Data are mainly presented as mean ± standard error of the mean (SEM) unless otherwise mentioned. All error bars represent SEM. Statistical analysis was performed with GraphPad Prism 6 (GraphPad Software) and Microsoft Excel

(Microsoft). Significant differences between data sets have been obtained with different tests as mentioned in the figure legends.

REFERENCES

Ganat, Y.M., Silbereis, J., Cave, C., Ngu, H., Anderson, G.M., Ohkubo, Y., Ment, L.R., and Vaccarino, F.M. (2006). Early postnatal astroglial cells produce multilineage precursors and neural stem cells in vivo. J Neurosci *26*, 8609-8621.

Joseph, N.M., He, S., Quintana, E., Kim, Y.G., Nunez, G., and Morrison, S.J. (2011). Enteric glia are multipotent in culture but primarily form glia in the adult rodent gut. J Clin Invest *121*, 3398-3411.

Laranjeira, C., Sandgren, K., Kessaris, N., Richardson, W., Potocnik, A., Vanden Berghe, P., and Pachnis, V. (2011). Glial cells in the mouse enteric nervous system can undergo neurogenesis in response to injury. J Clin Invest *121*, 3412-3424.

Matsuoka, T., Ahlberg, P.E., Kessaris, N., Iannarelli, P., Dennehy, U., Richardson, W.D., McMahon, A.P., and Koentges, G. (2005). Neural crest origins of the neck and shoulder. Nature *436*, 347-355.

Reikvam, D.H., Erofeev, A., Sandvik, A., Grcic, V., Jahnsen, F.L., Gaustad, P., McCoy, K.D., Macpherson, A.J., Meza-Zepeda, L.A., and Johansen, F.E. (2011). Depletion of murine intestinal microbiota: effects on gut mucosa and epithelial gene expression. PloS one *6*, e17996.

Reinhardt, C., Bergentall, M., Greiner, T.U., Schaffner, F., Ostergren-Lunden, G., Petersen, L.C., Ruf, W., and Backhed, F. (2012). Tissue factor and PAR1 promote microbiota-induced intestinal vascular remodelling. Nature *483*, 627-631.

Snippert, H.J., van der Flier, L.G., Sato, T., van Es, J.H., van den Born, M., Kroon-Veenboer, C., Barker, N., Klein, A.M., van Rheenen, J., Simons, B.D.*, et al.* (2010). Intestinal crypt homeostasis results from neutral competition between symmetrically dividing Lgr5 stem cells. Cell *143*, 134-144.

Srinivas, S., Watanabe, T., Lin, C.S., William, C.M., Tanabe, Y., Jessell, T.M., and Costantini, F. (2001). Cre reporter strains produced by targeted insertion of EYFP and ECFP into the ROSA26 locus. BMC Dev Biol *1*, 4.

Tasic, B., Miyamichi, K., Hippenmeyer, S., Dani, V.S., Zeng, H., Joo, W., Zong, H., Chen-Tsai, Y., and Luo, L. (2012). Extensions of MADM (mosaic analysis with double markers) in mice. PloS one *7*, e33332.

Zong, H., Espinosa, J.S., Su, H.H., Muzumdar, M.D., and Luo, L. (2005). Mosaic analysis with double markers in mice. Cell *121*, 479-492.
