# Supplementary figures and images for: Microbiota Controls the Homeostasis of Glial Cells in the Gut Lamina Propria
Source: Neuron. 2015 Jan 21;85(2):289–95. doi: 10.1016/j.neuron.2014.12.037 (PMC4306542; doi:10.1016/j.neuron.2014.12.037)

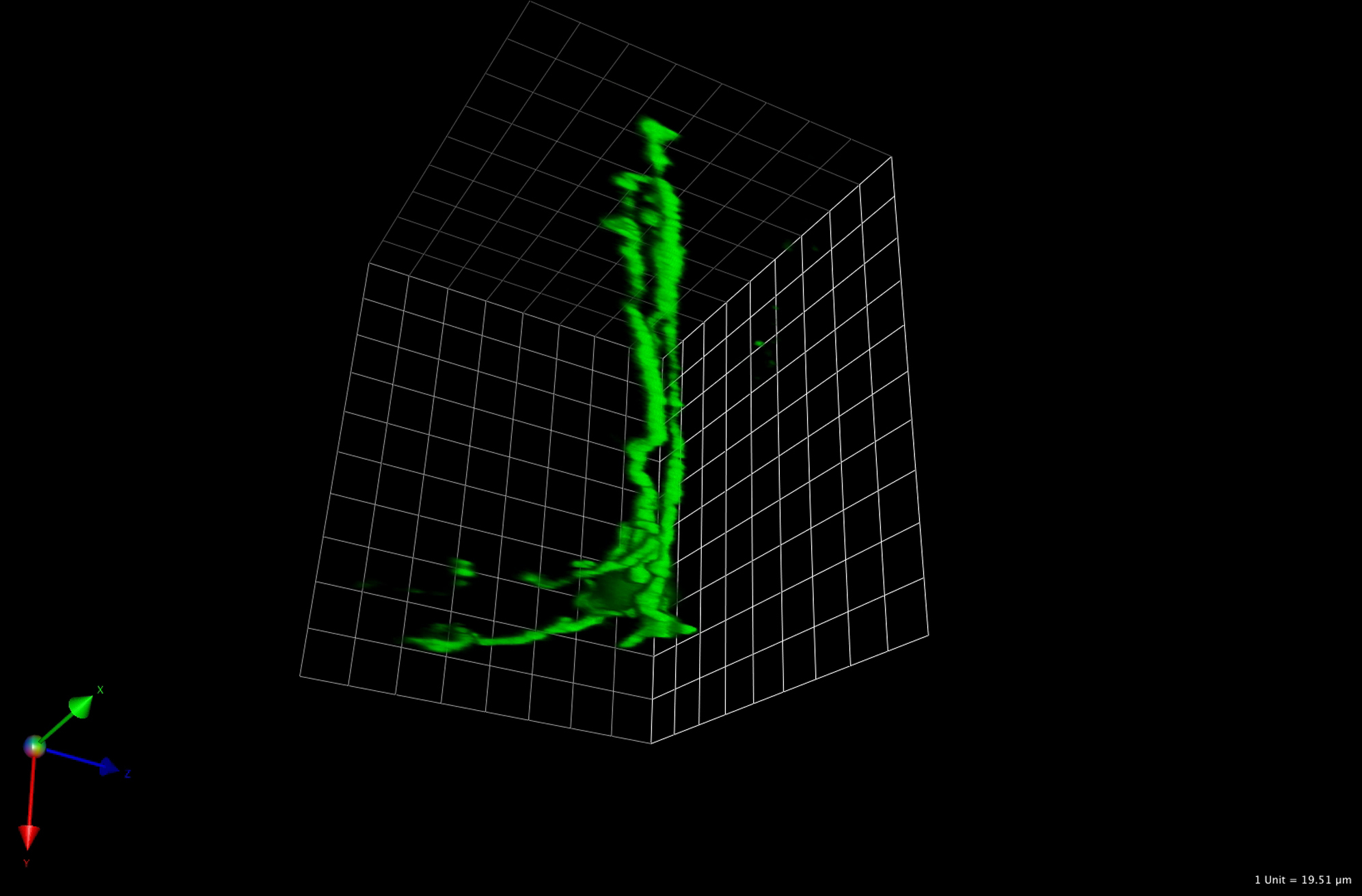

Supplement: Movie S1, Related to Figure 1. mEGCs Are Highly Branched — Shown is a surface-rendered reconstruction of confocal images of a single GFP+ glial cell (green) in a villus of Sox10::Cre;MADMGR/RG mice. [file mmc2.jpg]

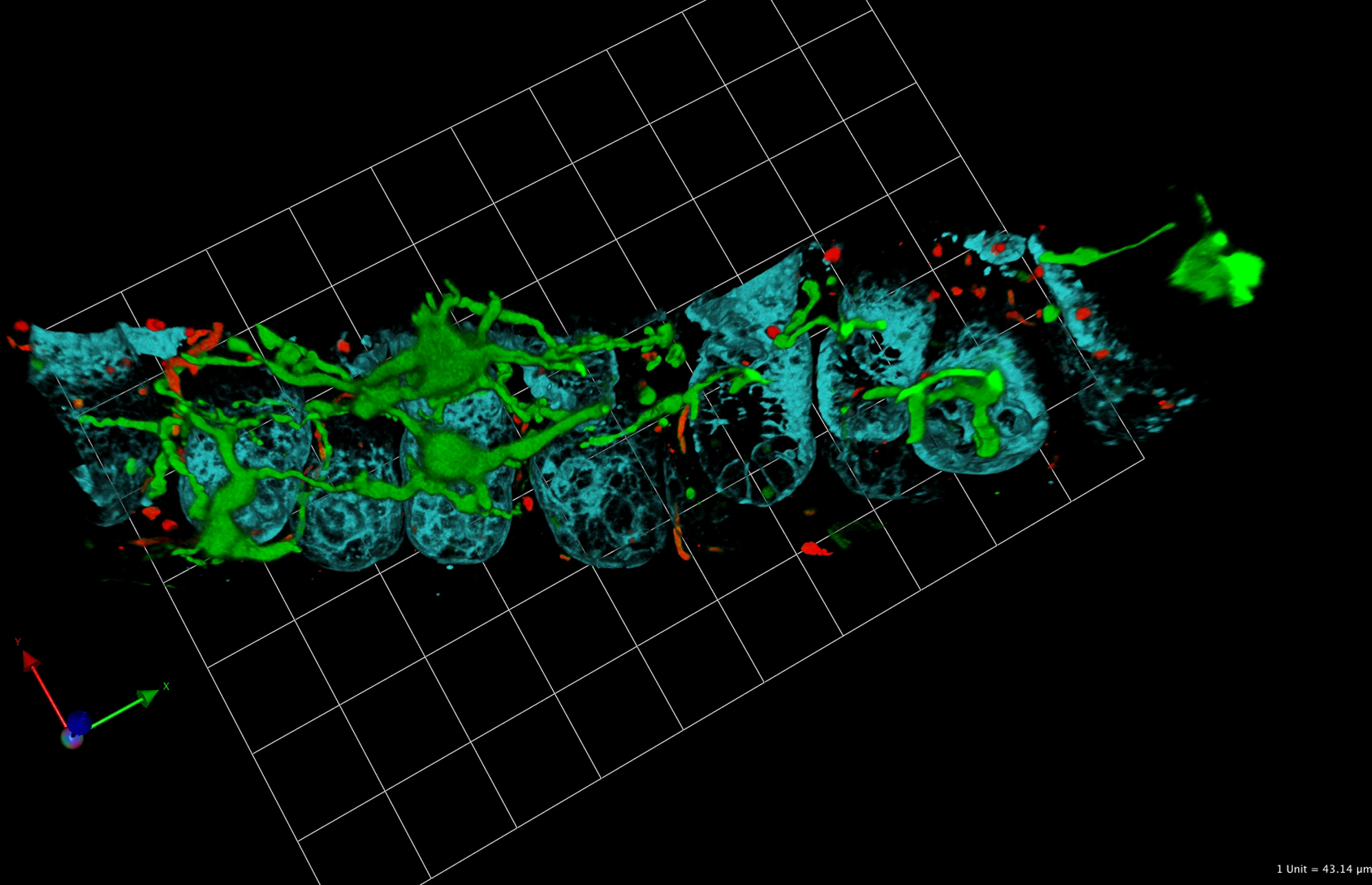

Supplement: Movie S2, Related to Figure 1. mEGCs Are Closely Associated with Crypts — Shown is a surface-rendered reconstruction of confocal images of crypt epithelial cells immunostained for A33 (cyan) in close association with GFP+ glial cell network (green) from the ileum of a Sox10::Cre;MADMGR/RG mouse. Parts of the vascular network are highlighted by Rhodamine dextran sulphate (red). [file mmc3.jpg]

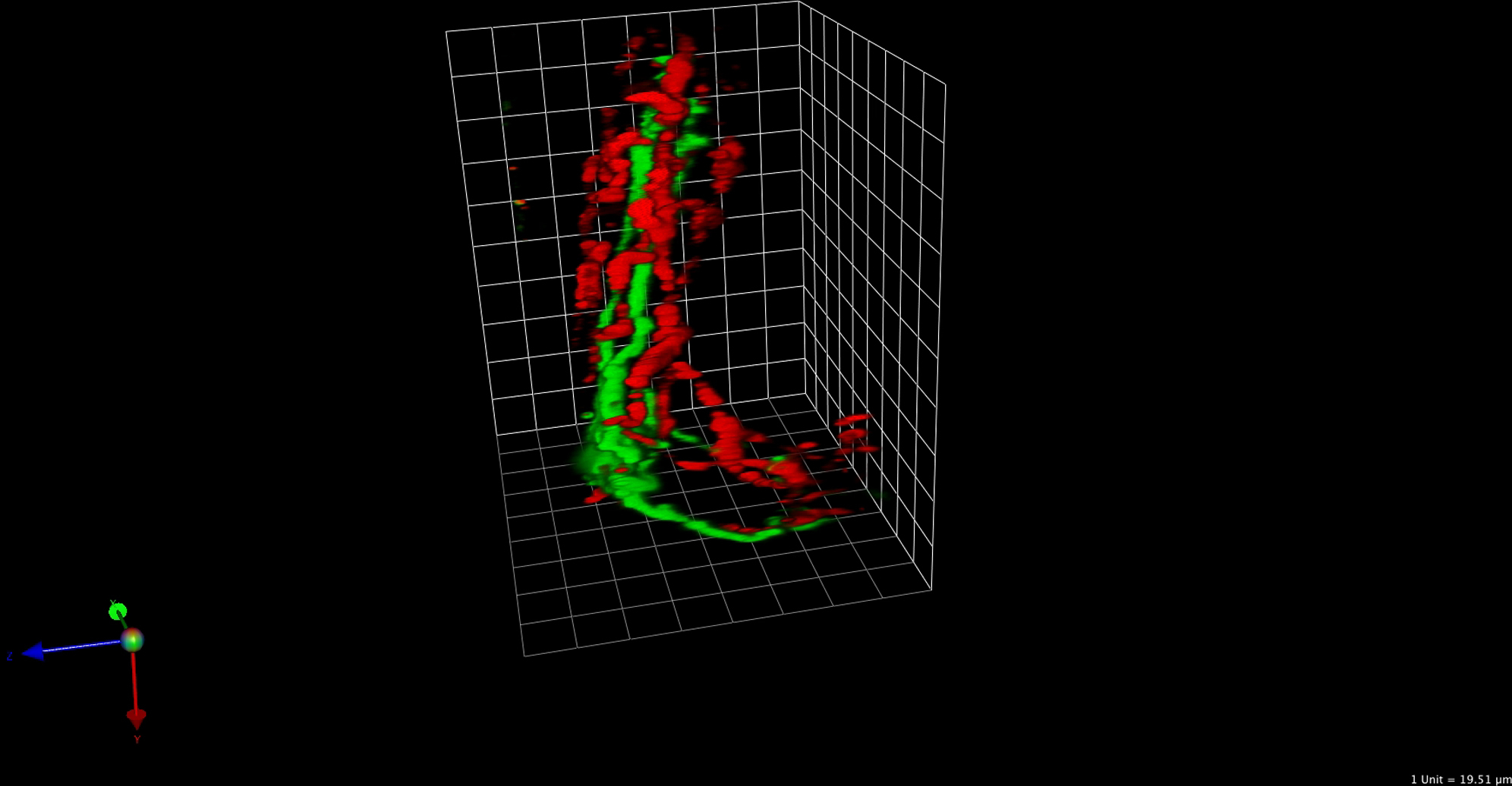

Supplement: Movie S3, Related to Figure 1. mEGCs Interact Closely with the Vascular Network of Villi — Shown is a surface-rendered reconstruction of confocal images of a GFP+ glial cell (green) and Rhodamine Dextran-labeled microvasculature (red) in a villus of a Sox10::Cre;MADMGR/RG mouse. [file mmc4.jpg]
